# Secreted hCLCA1 Is a Signaling Molecule That Activates Airway Macrophages

**DOI:** 10.1371/journal.pone.0083130

**Published:** 2013-12-12

**Authors:** John C. H. Ching, Liubov Lobanova, Matthew E. Loewen

**Affiliations:** Department of Veterinary Biomedical Sciences, University of Saskatchewan, Saskatoon, Saskatchewan, Canada; Lovelace Respiratory Research Institute, United States of America

## Abstract

The *CLCA* gene family produces both secreted and membrane-associated proteins that modulate ion-channel function, drive mucus production and have a poorly understood pleiotropic effect on airway inflammation. The primary up-regulated human CLCA ortholog in airway inflammation is hCLCA1. Here we show that this protein can activate airway macrophages, inducing them to express cytokines and to undertake a pivotal role in airway inflammation. In a U-937 airway macrophage–monocyte cell line, conditioned media from HEK 293 cells heterologously expressing hCLCA1 (with or without fetal bovine serum) increased the levels of pro-inflammatory cytokines (IL-1β, IL-6, TNF-α and IL-8). This effect was independent of the metalloprotease domain of hCLCA1. Primary porcine alveolar macrophages were similarly activated, demonstrating the effect was not cell line dependent. Similarly, immuno-purified hCLCA1 at physiologically relevant concentration of ~100 pg/mL was able to activate macrophages and induce pro-inflammatory response. This cytokine response increased with higher concentration of immuno-purified hCLCA1. These findings demonstrate the ability of hCLCA1 to function as a signaling molecule and activate macrophages, central regulators of airway inflammation.

## Introduction

*CLCA* genes (*CL* stands for chloride-channel modulating and *CA* for calcium-activated) are induced in airway epithelial cells by inflammation [1]. This induced expression often exceeds that of most other inflammatory mediators [2]. The gene products have a pleiotropic effect, generating secreted and membrane-associated proteins that increase mucus production, leukocyte infiltration and airway hyper-responsiveness, with single-nucleotide polymorphisms increasing asthma susceptibility as well [1-7]. Although CLCA proteins were originally identified as calcium activated chloride channels, we and others concluded that they only modulated channel pores (suggesting a signaling ability) [8-12]. How putative signaling ligands could cause a seemingly pleotropic effect on airway inflammation was unclear. One possibility was that CLCAs modified a central mediator of airway inflammation, such as the airway macrophage.

Airway macrophages are one of the major resident immune cell types responsible for lung defense [13]. How a macrophage is activated will determine its function [14]. “Classically” activated macrophages secrete high levels of pro-inflammatory cytokines (such as IL-8, IL-6 and IL-1β), enhancing their microbiocidal capacity by producing oxygen and nitrogen free radicals [14]. “Alternatively” activated macrophages secrete high levels of anti-inflammatory cytokines (such as IL-10 and IL-12), dampening the immune response and promoting wound healing [15]. Generally, macrophage activation has a receptor-driven signal transduction mechanism requiring activation of (cytoplasmic membrane) potassium and chloride channels to proceed [16-18]. The primary up-regulated human CLCA ortholog in human airway inflammation is hCLCA1. If this protein activates macrophages, the pleiotropic effect of *CLCA* genes can be explained.

In this report, we used a human monocyte cell line (U-937) and primary porcine alveolar macrophages to test whether the secreted form of hCLCA1 can activate macrophages. We also assessed the role of the autoproteolytic metalloprotease (hydrolase) domain of hCLCA1, which contains a zinc-reactive HEXXH motif that cleaves the protein into a large ~90 kD N-terminal and a small ~40 kD C-terminal fragment, in the macrophage activation process [19,20]. We found using progressively purified secreted hCLCA1 protein, that it possesses an intrinsic ability to signal and activate airway macrophages. This signaling property was independent of its hydrolase domain activity.

## Materials and Methods

### Cell culture and transfection

Human embryonic kidney cells (HEK293) were grown in DMEM-Glutamax medium (10566-016; Life Technologies) supplemented with 10% fetal bovine serum (FBS; 16000-044; Life Technologies) and 1% penicillin-streptomycin (pen-strep; 15140-122; Life Technologies) at 37 °C in a humidified atmosphere with 5% CO_2_. Cells were seeded and transfected in six-well plates with the vectors pIRES2-EGFP, wild-type pIRES2-EGFP-hCLCA1 or hydrolase-inactive E157Q mutant pIRES2-EGFP-hCLCA1 (GenScript) using the transfection reagent FuGENE HD (E2311; Promega) according to manufacturers’ protocols.

Human monocytes (U-937 cell line; CRL1593.2; ATCC) were grown in RPMI-1640 medium (SH3025502; Thermo Scientifics) supplemented with 10% heat inactivated FBS and 1% pen-strep at 37 °C in a humidified atmosphere with 5% CO_2_. Porcine alveolar macrophages were grown in RPMI-1640 medium supplemented with 20% heat inactivated FBS and 1% pen-strep at 37 °C in a humidified atmosphere with 5% CO_2_. 

### Media collection, immunoprecipitation and protein concentration determination

At Day 2 post-transfection, conditioned FBS-containing medium was collected. Conditioned FBS-free medium was collected at Day 3 (after replacing the initial HEK293 medium with FBS-free DMEM at Day 2). Macromolecules in the collected media were concentrated using Amicon Ultra-15 Centrifugal Filter Units (UFC903008; EMD Millipore). The concentrations of the proteins were determined using a Bradford protein assay (500-0201; Bio-rad)

Conditioned FBS-free hCLCA1 and FBS-free eGFP macromolecule samples were immunoprecipitated with a Pierce Crosslink Magnetic IP/Co-IP Kit (88805; Thermo Scientific) using hCLCA1-N14 antibody (sc-46866; Santa Cruz) according to manufacturers’ protocols. To improve the yield of the immunoprecipitation, we increased: the antibody amount used in the antibody coupling step to 8 μg from 5 μg, the antibody coupling time to 30 minutes from 15 minutes, and the sample incubation time to 1.5 h from 1 h. The concentration of the immunoprecipitated hCLCA1 protein was determined from a standard curve generated using a 2-fold dilution series of lysozyme (L-6876; Sigma-Aldrich) on a silver stained SDS-PAGE gel. The concentrations of lysozyme used in the standard curve were 100 pg/μL, 50 pg/μL, 25 pg/μL, 12.5 pg/μL, 6.25 pg/μL, and 3.125 pg/μL.

### Monocyte differentiation and activation

Monocyte cells were seeded in each well (1.3×10^6^ to 1.5×10^6^ cells/well) in a 6-well plate and differentiated into macrophages with 0.1 nM phorbol-12-myristate-13-acetate (P8139; Sigma-Aldrich) in FBS-free RPMI-1640 medium for 24 h. The cells were washed 3 times with supplemented FBS-free RPMI-1640 medium and incubated in supplemented RPMI-1640 media containing 3, 6 or 10% FBS. In the conditioned FBS-containing medium experiment, 0.1, 1 or 10 mg/mL of eGFP or hCLCA1 was added to macrophages in 10% FBS growth medium. In conditioned FBS-free medium experiment, 66.7 μg/mL or 200.0 μg/mL of eGFP or hCLCA1 was added to macrophages in 3, 6, or 10% FBS growth medium to determine optimal FBS concentration; and 3.3 μg/mL, 16.7 μg/mL or 33.3 μg/mL of hCLCA1 was added to macrophages in 6% FBS growth medium to determine the dose response. In a further experiment, 45 μL of immuno-purified hCLCA1 (93.3 pg/mL or 141.7 pg/mL) or 45 μL of control (immunoprecipitation of eGFP using hCLCA1-N14 antibody) were added and incubated in 6% FBS growth medium for 24 h or 48 h.

### SDS-PAGE and Western Blot Analysis

Cell lysates were collected using M-PER mammalian protein extraction reagent (78503; Thermo Scientific) with the addition of Halt protease and phosphatase inhibitor cocktail (78440; Thermo Scientific). The samples (cell lysates or media) were boiled in 2x denaturing buffer (20% glycerol, 4% SDS, 125mM Tris pH 6.8, 0.3mM bromophenol blue) and analyzed by 10% or 12% SDS-PAGE. The SDS-PAGE gel was stained with coomassie blue stain (staining – 45% methanol, 10% glacial acetic acid, 45% water, 3g/L Coomassie Brilliant Blue R250; destaining – 20% methanol, 10% glacial acetic acid, 70% water) and a Pierce Color Silver Stain Kit (24597; Thermo Scientific) according to manufacturers’ protocols. Densitometry was performed using a ChemiDoc MP System (170-8280; Bio-rad)

For western blot analysis, proteins were electroblotted onto PVDF membrane (RPN303LFP; GE Healthcare Life Sciences) with transfer buffer (25mM Tris, 192mM glycine, 20% methanol). Membranes were blocked overnight at 4 °C with 5% bovine serum albumin in PBST buffer (137mM NaCl, 2.6mM KCl, 1.5mM KH_2_PO_4_, 8.1mM Na_2_HPO_4_, 0.1% Tween-20; Sigma-Aldrich) and subsequently probed for 2 h at room temperature with primary antibodies in PBST. The membranes were then incubated for 1 h at room temperature with secondary antibodies in PBST. Proteins were detected and analyzed using Typhoon Trio and ImageQuant TL system (63005583; GE healthcare Life Sciences). Densitometry analysis of intracellular IL-1β protein was normalized to GAPDH in each sample as previously described [21,22]. The primary antibodies used were hCLCA1 (N-14; sc-46866; Santa Cruz), GAPDH (FL-335; sc-25778; Santa Cruz), IL-1β (H-153; sc-7884; Santa Cruz), and GAPDH (MAB374; EMD Millipore). The secondary antibodies used were Alexa Fluor 488 Donkey anti-Goat IgG antibody (A11055; Life Technologies), ECL Plex Goat anti-Mouse IgG-Cy5 antibody (PA45009; Amersham Biosciences), and DyLight 488 conjugate Goat anti-Rabbit IgG antibody (35552; Thermo Scientific).

### Bio-Plex Suspension Array System

After 48 h activation with immuno-purified proteins, macrophage medium was collected and analyzed for cytokine levels using Bio-Plex Suspension Array System (171-000201; Bio-rad) according to the manufacturers’ protocols. The Bio-Plex Pro Human Cytokine 8-plex Assay (M50-000007A; Bio-rad) we used included the following cytokines: IL-2, IL-4, IL-6, IL-8, IL-10, GM-CSF, IFN-γ, and TNF-α.

### RNA isolation and real-time quantitative PCR

For macrophages activated in FBS-containing media, RNA was extracted after 1, 2, 4, 6, 12 and 24 h using TRIzol Reagent (15596018; Life Technologies) according to manufacturers’ protocols. For macrophages activated in FBS-free media, RNA was extracted after 24 h. For macrophages activated with immunoprecipitated proteins, RNA was extracted after 24 or 48 h. The collected RNA was analyzed with a GoTaq 2-Step RT-qPCR system (A6010; Promega) and Mx3005P real-time PCR machine (401514; Agilent). cDNA of each sample was measured in duplicates in Mx3005P real-time qPCR machine, and the average *C*_T_ (cycle threshold) value was used to calculate the fold difference of each gene. Human primers were designed for GAPDH, TNF-α, IL-12a, IL-8, IL-1β, IL-6 and IL-10; and porcine primers were designed for NADH, TNF-α, IL-12a, IL-8, IL-1β, IL-6 and IL-10 (Table 1). GAPDH and NADH were used as the reference genes for analysis.

**Table 1:** Primers used in RT-qPCR experiments.

**Human qPCR Primers**		
**Gene Name**	**Forward Primers** (**5′ 3′**)	**Reverse Primers** (**5′ 3′**)
**GAPDH**	CAAGGTCATCCATGACAACTTTG	GGGCCATCCACAGTCTTCTG
**TNF-α**	TGCTGCACTTTGGAGTGATCG	TGCTACAACATGGGCTACAGG
**IL-12a**	CAGTGGAGGCCTGTTTACCATTG	TACTACTAAGGCACAGGGCCATC
**IL-8**	TCTCTTGGCAGCCTTCCTGATTTC	ATTTCTGTGTTGGCGCAGTGTG
**IL-1β**	GCTGATGGCCCTAAACAGATG	TGTAGTGGTGGTCGGAGATTC
**IL-6**	AGCCACTCACCTCTTCAGAAC	GTGCCTCTTTGCTGCTTTCAC
**IL-10**	AAGCTGAGAACCAAGACCCAGACA	AAAGGCATTCTTCACCTGCTCCAC
**Porcine qPCR Primers**		
**Gene Name**	**Forward Primers**	**Reverse Primers**
**NADH**	TCATCGGGGCCCTACGAGCA	GGCGAAAGGTCCGGCTGCAT
**TNF-α**	ACGCTCTTCTGCCTACTGCACTTC	TCCCTCGGCTTTGACATTGGCTAC
**IL-12a**	CCACTTGAACTAGCCACGAATGAG	AGATACTGCTAAGGCACAGGGTTG
**IL-8**	AGGACCAGAGCCAGGAAGAGAC	CTTGCCAGAACTGCAGCCTCAC
**IL-1β**	CTCCAGCCAGTCTTCATTGTTCAG	GTTGTCACCGTAGTTAGCCATCAC
**IL-6**	CCAATCTGGGTTCAATCAGGAGAC	CAGCCTCGACATTTCCCTTATTGC
**IL-10**	AAGACGTAATGCCGAAGGCAGAGA	TGCTAAAGGCACTCTTCACCTCCT

### Ethics Statement

Obtaining and use of animal tissue was specifically approved for this study by the University of Saskatchewan’s Institutional Animal Care and Use Committee (IACUC) Permit # 20120100.

### Porcine alveolar macrophage isolation protocol

A solution of 100 mL of 0.1 M PBS supplemented with 1% pen-strep and 1x antibiotic-antimycotic (15240112; Life Technologies) was injected into porcine lungs. The lungs were obtained with specific permission for use in this study from a local federally inspected abattoir (Friesen's Meat Processing, Warman, SK). Lung lavage fluid was collected and filtered through 70 μm cell strainer (352350; Corning). The filtered cells were subjected to repeated centrifugation at 400*g* for 10 min at 4 °C and washed with 20 mL 1.0 M PBS (supplemented with 1% pen-strep and 1x antibiotic-antimycotic), 2–3 mL of erythrocyte lysing solution (for 20 s), 45 mL of 1.0 M PBS and then 25 mL of 1.0 M PBS. The pelleted cells were subsequently re-suspended in culture medium (RPMI-1640 with 20% heat-inactivated FBS, 1% pen-strep and 1x antibiotic-antimycotic). The cells were counted using trypan blue (15250061; Life Technologies), and 1.5×10^7^ cells/mL were frozen down using culture medium with 10% DMSO.

### Porcine alveolar macrophage stimulation

A total of 1.5×10^7^ cells were thawed at 37 °C, added to 8 mL warm culture medium and centrifuged at 500*g* for 5 min. The cells were then re-suspended, and 1.5×10^7^ cells were seeded in each well of a six-well plate with 3 mL of culture medium in each well. The plates were incubated 15–18 h at 37 °C under a humidified 5% CO_2_ atmosphere. The cells were washed 2 times with culture medium and incubated in 3 mL culture medium. To activate the macrophages, 66.7 µg/mL, 200 µg/mL or 1000 µg/mL of eGFP or hCLCA1 in FBS-free medium was added.

### Efficiency and fold difference calculations

Dilution series from 1×10^0^-fold to 1×10^−5^-fold of cDNA were used to determine the primer efficiency. The *C*_T_ value obtained in each dilution was used to generate a linear plot of *C*_T_ vs. log copies. The efficiency of the primer set was determined with the equation *Eff* = 10^(−1/slope)^. The fold difference between hCLCA1- and eGFP-activated samples was determined using an efficiency-corrected calculation with eGFP-activated macrophage served as control and GAPDH or pNADH served as reference gene [23]:

$$ratio={\left(Eff_{\text{target}}\right)}^{\Delta C_{\text{T},\text{ target}}\left(\text{Mean control }-\text{ Mean sample}\right)}/{\left(Eff_{\text{ref}}\right)}^{\Delta C_{\text{T, ref}}\left(\text{Mean control }-\text{ Mean sample}\right)}$$

### Statistics

All data are expressed as means ± standard error of the mean (SEM). Each biological replicate was a result of an individual transfection paired with an eGFP transfection. Each biological replicate was performed on different days. Fold differences were calculated by comparing hCLCA1-activated macrophage to its paired eGFP-activated macrophage control. The normality test was done using Shaprio-Wilk test, and the fold difference values of RT-qPCR and Bio-Plex assay were analyzed using Kruskal-Wallis one-way analysis of variance test with Conover-Inman test. Friedman two-way analysis of variance was performed to compare the fold difference of mRNA expression between groups (time, wild type hCLCA1 vs. mutant hCLCA1, concentration, etc). All western blot data was normalized to the appropriate controls, and each western blot was a result of an individual biological replicate. One-way t-tests were performed with the mean set as 1 [24,25]. Significance was determined at *p* < 0.05. 

## Results

### Response of the U-937 macrophage cell line to FBS-containing hCLCA1 medium

**Figure 1:**
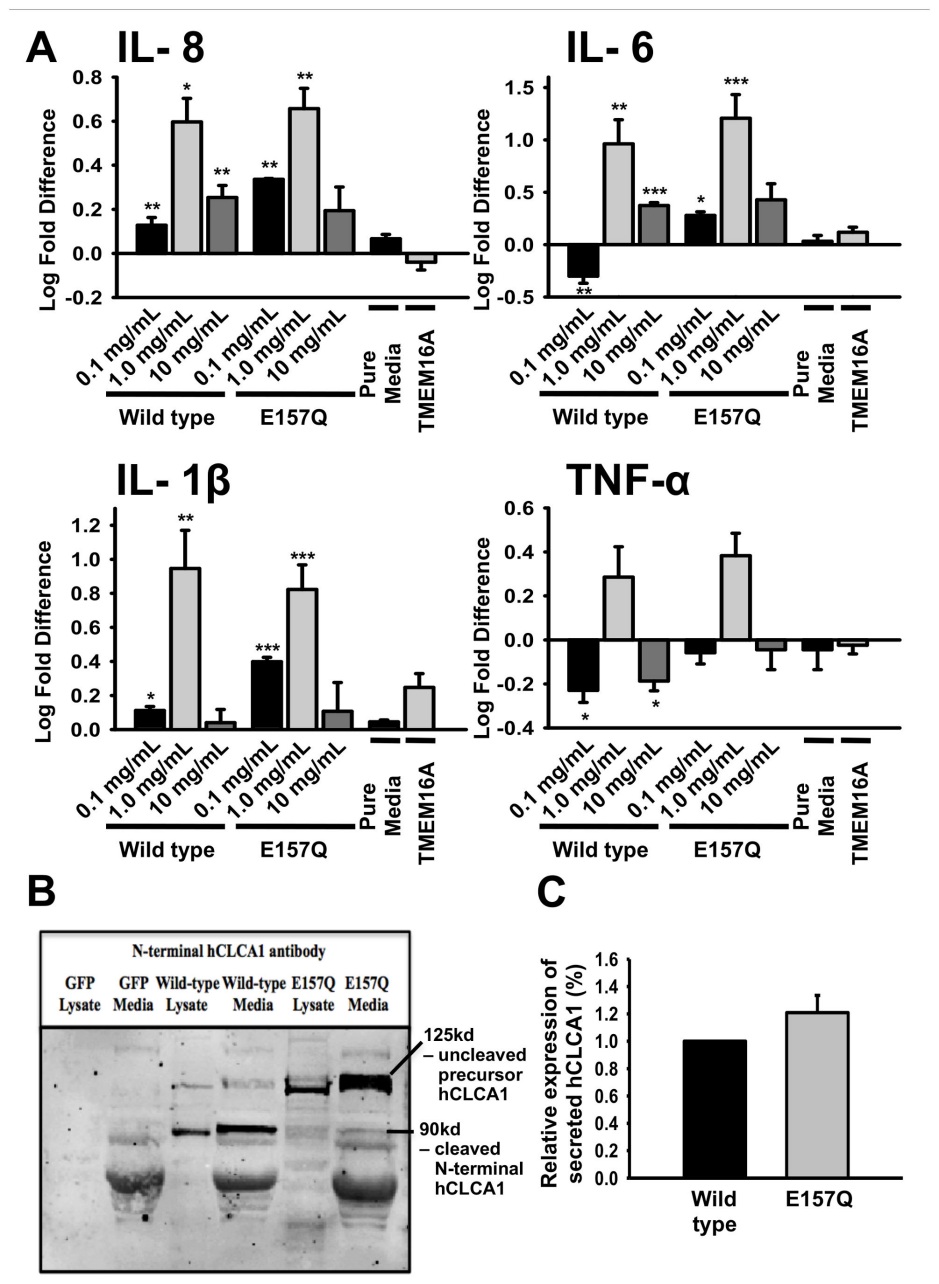
Conditioned FBS-containing hCLCA1 medium shows dose-dependent effect. (**A**) Macrophages were activated for 24 h with 0.1 mg/mL, 1.0 mg/mL, or 10 mg/mL of FBS-containing stimulants (eGFP, wild-type hCLCA1, E157Q mutant hCLCA1, 1.0 mg/mL pure media, or 1.0 mg/mL TMEM16A). Dose-dependent cytokine mRNA expression by macrophages was quantified through RT-qPCR. The fold difference at each condition was compared against eGFP (the control) of the same concentration. Results were presented as the means of 3 samples ± SEM. Each sample was a result of an individual transfection paired with an eGFP transfection. Significant fold differences from corresponding control values (eGFP of the same concentration) are indicated by * (*p* < 0.05), ** (*p* < 0.005) or *** (*p* < 0.001). (**B**) Representative western blot image of eGFP- and hCLCA1-transfected HEK-293 cell lysates and media using hCLCA1 N-terminal antibody. Both precursor and N-terminal hCLCA1 products were secreted into the extracellular space. However, there was a higher proportion of cleaved product than precursor in the wild-type hCLCA1 lysate and medium. (**C**) Relative protein expressions of secreted wild-type and secreted E157Q mutant hCLCA1 were compared using densitometry (the secreted E157Q mutant hCLCA1 was normalized to the secreted wild-type hCLCA1 in each sample). No statistical significant difference was found between the protein levels of wild-type and E157Q mutant hCLCA1 in the medium. Wild-type hCLCA1 included 125 kd uncleaved precursor and 90kd cleaved N-terminal hCLCA1, while E157Q mutant hCLCA1 only included 125kd uncleaved precursor hCLCA1. Results were presented as the means of 3 samples ± SEM.

**Figure 2:**
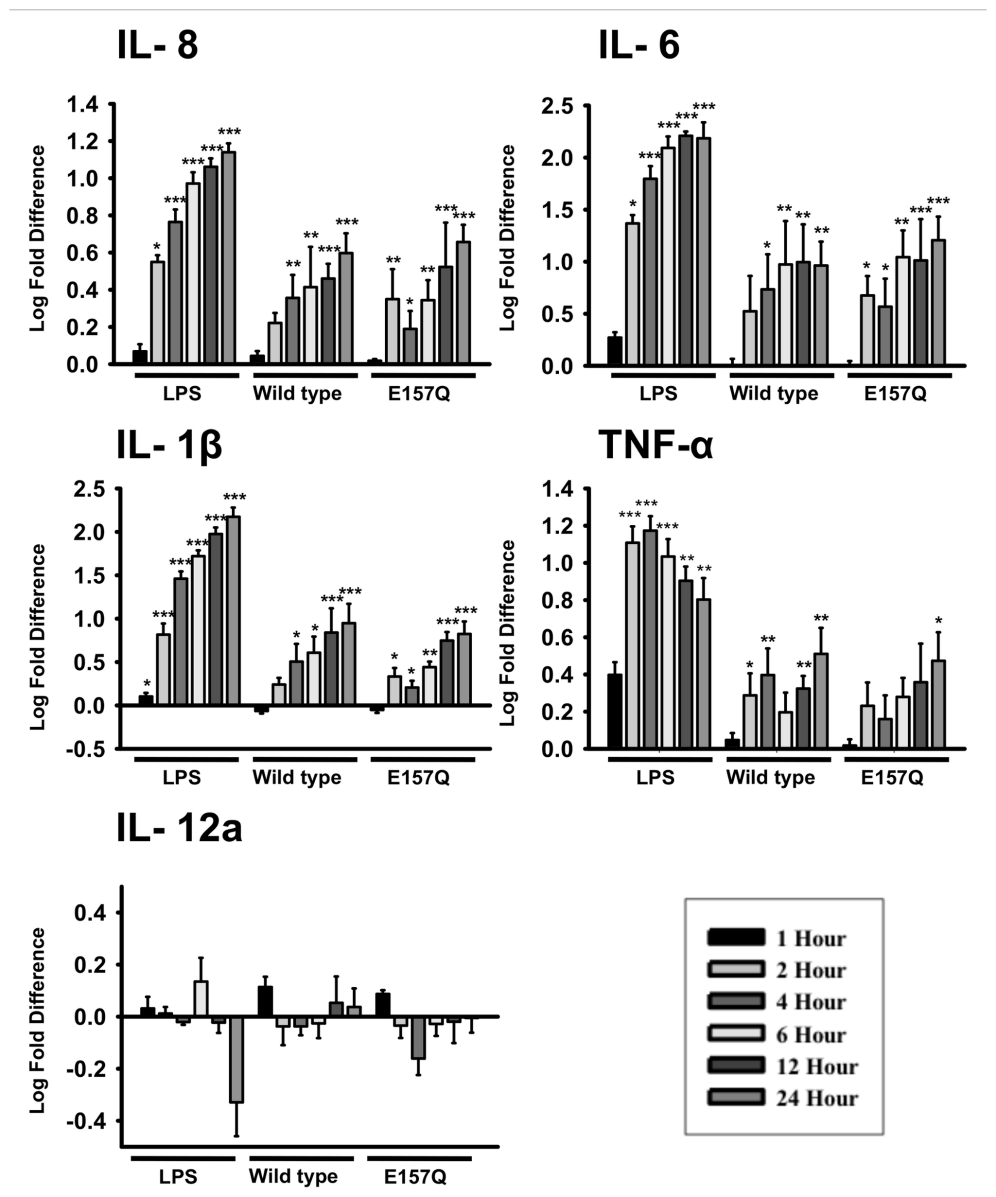
Conditioned FBS-containing hCLCA1 medium shows time-dependent effect. Macrophages were activated with 1.0 μg/mL LPS, 1.0 mg/mL FBS-containing wild-type hCLCA1, E157Q mutant hCLCA1 or eGFP medium. Time-dependent (1, 2, 4, 6, 12, 24 h) cytokine mRNA expression by macrophages was quantified through RT-qPCR. The fold difference at each condition was compared against eGFP (the control) of the same condition. Results were the means of 4 samples ± SEM. Each sample was a result of an individual transfection paired with an eGFP transfection. Significant fold differences from the corresponding control values (eGFP of the same concentration) are indicated by * (*p* < 0.05), ** (*p* < 0.005) or *** (*p* < 0.001).

The activation of macrophages by secreted hCLCA1 was investigated using the U-937 macrophage cell line treated with conditioned FBS-containing medium from HEK293 cells heterologously expressing hCLCA1 or eGFP. Macrophage activation was demonstrated by expression of the pro-inflammatory cytokines IL-8, IL-6, IL-1β and TNF-α (Figure 1A). The presence of secreted hCLCA1 in the medium was confirmed by western blot analysis (Figure 1B). There was more cleaved hCLCA1 product than the hCLCA1 precursor present in both wild-type hCLCA1 lysate and medium, whereas, E157Q hydrolase-inactive hCLCA1 had only the precursor present. Densitometry analysis showed that there was no significant difference between the expression of wild-type hCLCA1 and E157Q mutant hCLCA1 in medium (Figure 1C). In a log-scale dose test from 0.1 mg/mL to 10 mg/mL of total protein from the conditioned medium, the strongest response for these cytokines was obtained at 1 mg/mL (Figure 1A). Concentrated pure medium and conditioned medium from HEK293 cells expressing the large protein TMEM16A (tested as an additional control) was found to cause no significant activation (Figure 1A). At the optimized dose of 1 mg/mL, the expression of IL-8, IL-6, IL-1β and TNF-α in response to wild-type hCLCA1 and its hydrolase-inactive E157Q mutant was followed over 24 h (Figure 2); this test revealed no difference in the mRNA expression between E157Q and wild-type hCLCA1-activated macrophages. Generally, the longer the macrophages were incubated with hCLCA1, the higher the activation (Figure 2). Lipopolysaccharide (LPS), a potent endotoxin that activates through toll-like receptor 4, was used as a positive control and produced a similar time-dependent activation.

### Dose response of the U-937 cell line to FBS-free conditioned hCLCA1 medium

**Figure 3:**
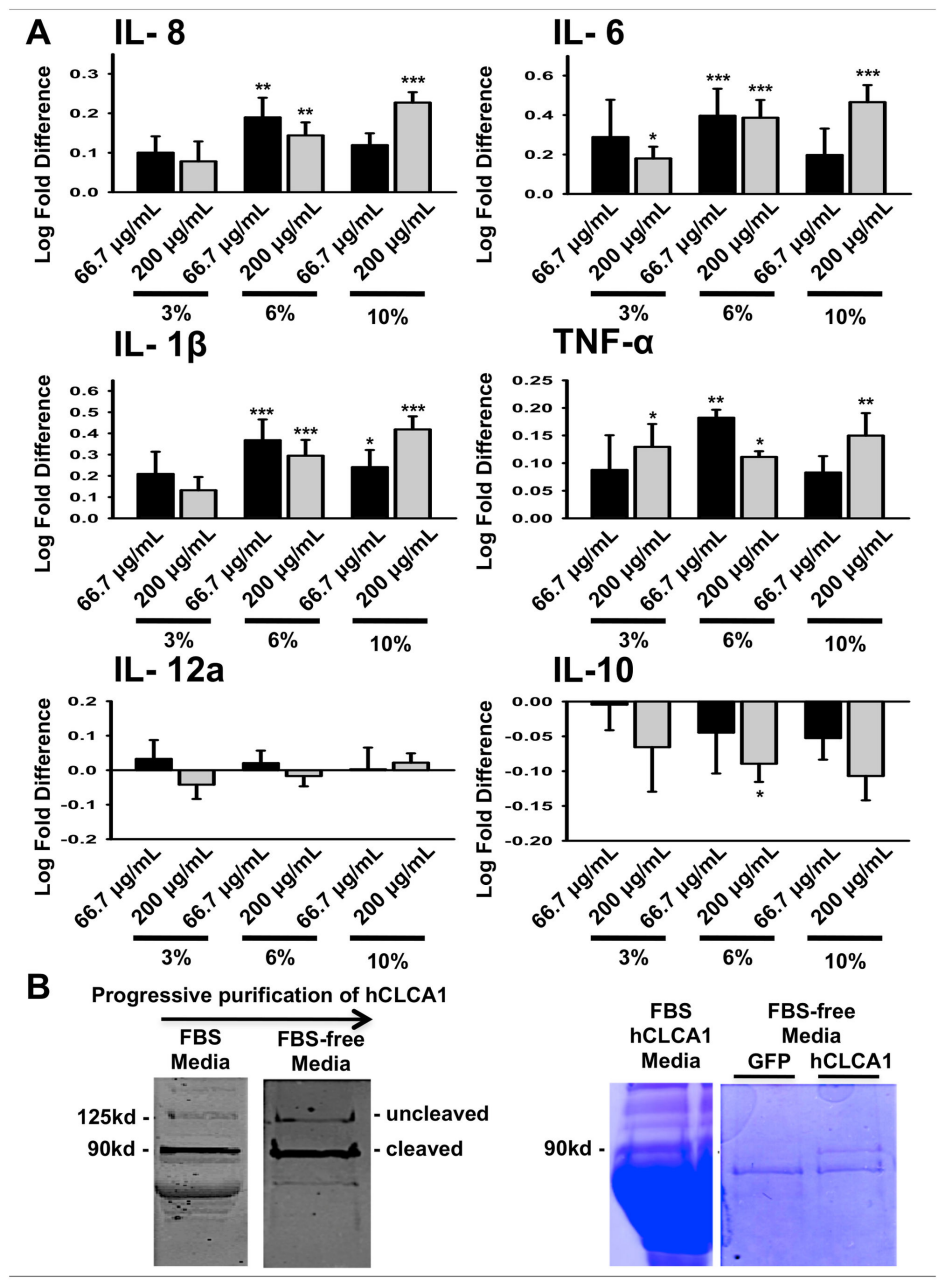
6% FBS is determined to be the optimal FBS % in growth medium to activate macrophages. (**A**) U-937 macrophage cells were activated for 24 h using 66.7 μg/mL or 200 μg/mL of FBS-free eGFP medium or FBS-free wild-type hCLCA1 medium with 3%, 6%, or 10% FBS growth medium. IL-8, IL-6, IL-1β, TNF-α, IL-12a and IL-10 were quantified by their mRNA expression using RT-qPCR. The fold difference at each concentration was compared against eGFP (the control) at the same concentration. Results were the means of 5 samples ± SEM. Each sample was a result of an individual transfection paired with an eGFP transfection. Significant fold differences from corresponding control values (eGFP of the same concentration) are indicated by * (*p* < 0.05), ** (*p* < 0.005) or *** (*p* < 0.001). (**B**) Representative western blot analysis probing against hCLCA1 N-terminal antibody was utilized in conjunction with Coomassie gel staining to compare the total protein contents between FBS-containing hCLCA1 medium and FBS-free hCLCA1 medium. Both western blot and coomassie gel staining showed that hCLCA1 was one of the major secreted molecules, and there was less impurities in the FBS-free hCLCA1 medium without a major loss in hCLCA1 protein.

**Figure 4:**
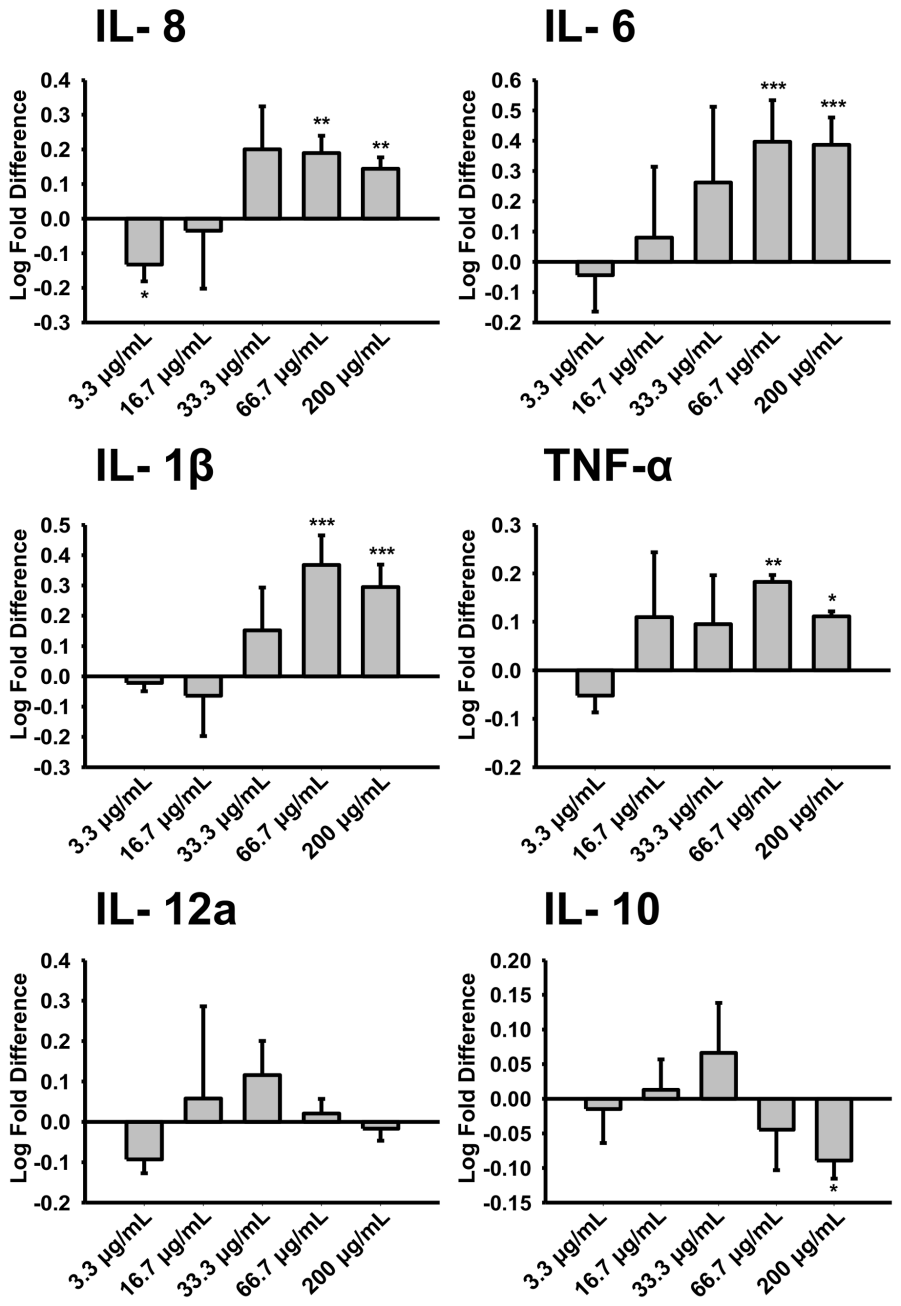
Conditioned FBS-free hCLCA1 medium shows a dose-dependent effect. U-937 macrophages were activated for 24 h using 3.3 μg/mL, 16.7 μg/mL, 33.3 μg/mL, 66.7 μg/mL, or 200 μg/mL of FBS-free wild-type hCLCA1 medium or FBS-free eGFP medium in 6% FBS growth medium. IL-8, IL-6, IL-1β, TNF-α, IL-12a and IL-10 were quantified by their mRNA expression using RT-qPCR. The fold difference at each concentration was compared against eGFP (the control) at the same concentration. Results were the means of 5 samples ± SEM. Each sample was a result of an individual transfection paired with an eGFP transfection. Significant fold differences from corresponding control values (eGFP of the same concentration) are indicated by * (*p* < 0.05), ** (*p* < 0.005) or *** (*p* < 0.001).

The macrophage growth medium for the dose response experiment was supplemented to the optimized concentration of 6% FBS, as determined by cytokine response (Figure 3A). An appropriate FBS concentration was essential for macrophage growth and activation. The expression of hCLCA1 was confirmed by western blot (Figure 3B). Comparing between the FBS-containing medium and FBS-free medium, both western blot and coomassie gel staining showed that there was significantly less impurities in the FBS-free medium (Figure 3B). The dose effect of conditioned FBS-free hCLCA1-containing medium on macrophage activation was carried out to eliminate any effects of FBS in the FBS-containing hCLCA1 medium (Figure 4). The concentration of total secreted protein in the medium used in Figure 1 was estimated to be between 50 and 200 µg/mL. Therefore, concentrations of FBS-free hCLCA1 medium ranging from 3.3 μg/mL to 200 μg/mL were used for the macrophage activation dose response test, in which 66.7 μg/mL of FBS-free hCLCA1 medium was found to provide the maximum increase in the expression of IL-8, IL-6, and IL-1β; conversely, 200 μg/mL of FBS-free hCLCA1 medium caused a significant decrease in the anti-inflammatory cytokine IL-10 (Figure 4). The level of IL-12a never responded significantly and was generally just at the level of detection (Figure 4). 

### Activation of primary porcine macrophages by hCLCA1

**Figure 5:**
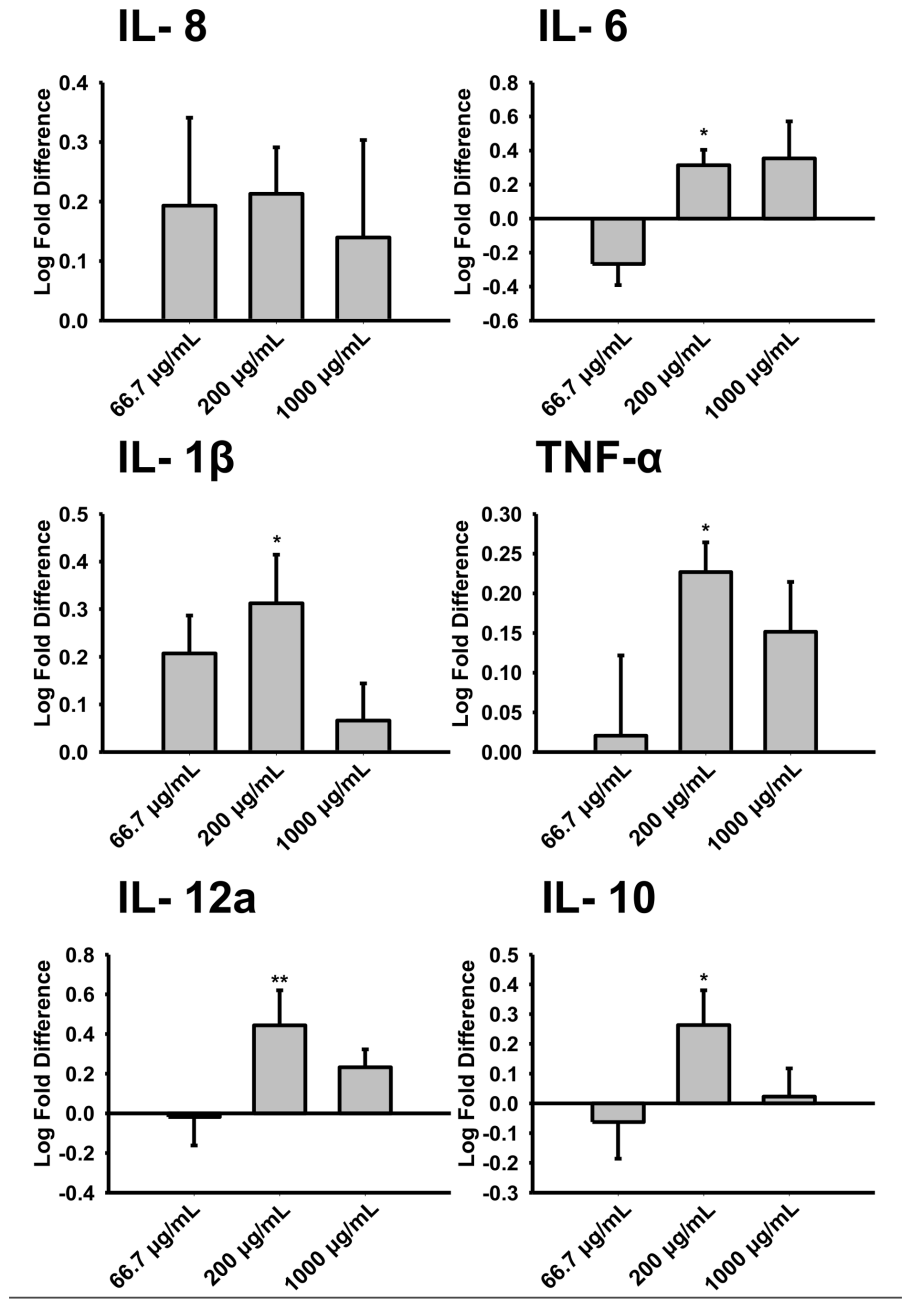
Activation of porcine alveolar macrophages with hCLCA1. Porcine alveolar macrophages were activated with different concentrations (66.7 μg/mL, 200 μg/mL or 1000 μg/mL) of FBS-free eGFP medium or FBS-free wild-type hCLCA1 medium. IL-8, IL-6, IL-1β, TNF-α, IL-12a and IL-10 were quantified by their mRNA expression using RT-qPCR. The fold difference at each concentration was compared against eGFP (the control) at the same concentration. Results were the means of 7 samples ± SEM. Each sample was a result of an individual transfection paired with an eGFP transfection. Significant fold differences from corresponding control values (eGFP of the same concentration) are indicated by * (*p* < 0.05), ** (*p* < 0.005) or *** (*p* < 0.001).

To confirm that the effect of hCLCA1 was not specific to the U-937 cell line, isolated porcine alveolar macrophages were tested with FBS-free hCLCA1 or eGFP medium. Activation by hCLCA1 was observed in 20% FBS medium, a standard growth condition for isolated porcine macrophages (Figure 5). However, 66.7 µg/mL of FBS-free hCLCA1 medium activated only IL-1β; 200 µg/mL of FBS-free hCLCA1 medium was required to increase most of the pro-inflammatory cytokines (including TNF-α, IL-8, IL- 6 and IL-1β). At 1000 µg/mL FBS-free hCLCA1 medium, however, the pro-inflammatory response was reduced.

### Activation of the U-937 cell line by immuno-purified hCLCA1

**Figure 6:**
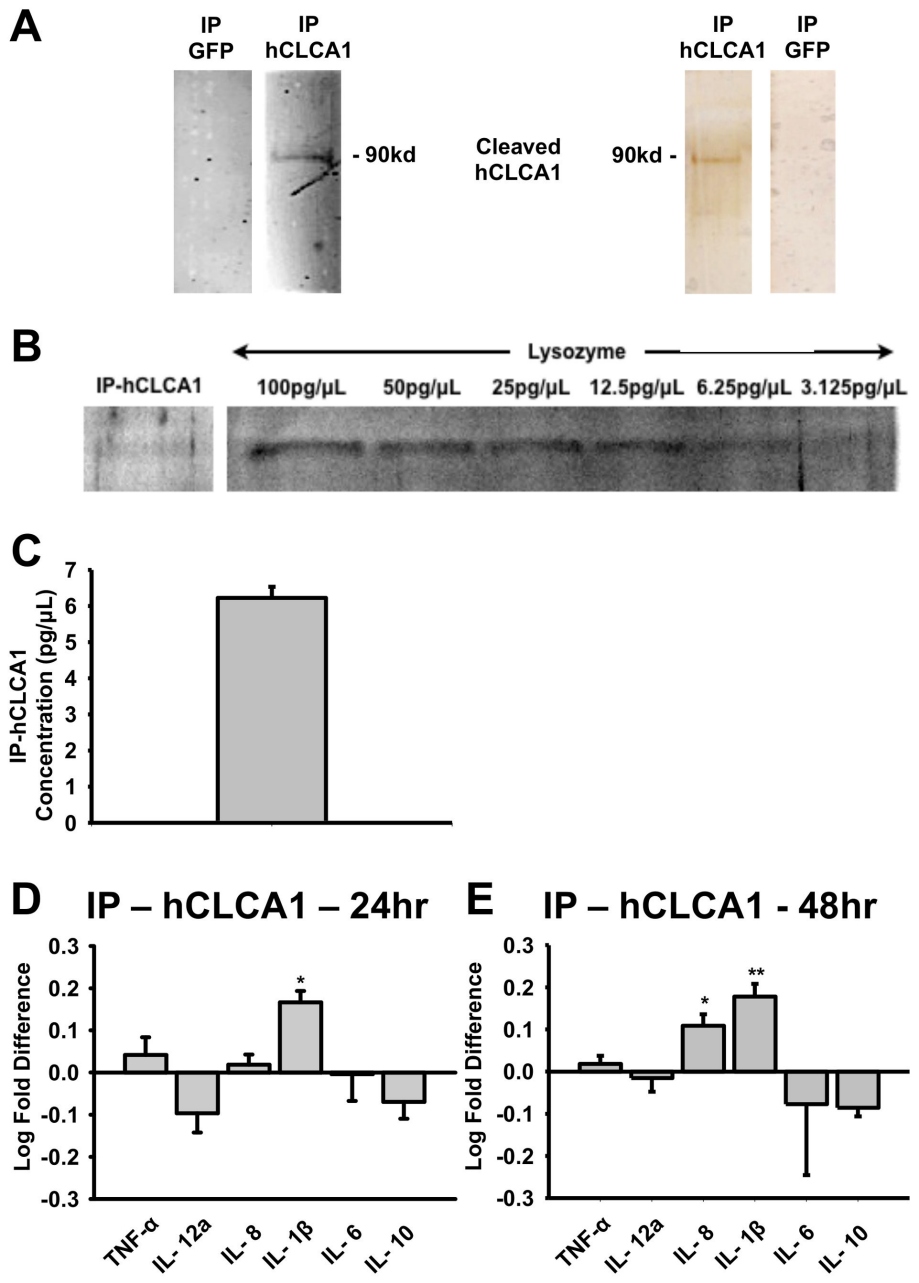
Activation of macrophages with immuno-purified hCLCA1. **A**) Western blot analysis of immuno-purified hCLCA1 and eGFP using hCLCA1 N-terminal antibody; and silver stained acrylamide gel containing immuno-purified hCLCA1 and eGFP. (**B**) Using a standard curve generated from a 2-fold dilution series of lysozyme on a silver stained SDS-PAGE gel, (**C**) immuno-purified hCLCA1 was determined to be 6.225 ± 0.307 pg/μL. Result was presented as the mean of 3 samples ± SEM. The mRNA expression of cytokines in macrophages stimulated with immuno-purified hCLCA1 for (**D**) 24 h and (**E**) 48 h were quantified using RT-qPCR. The fold difference was calculated against the corresponding control (immunoprecipitation of eGFP using hCLCA1-N14 antibody). Results for (**D**) and (**E**) were presented as the means of 10 samples ± SEM. Each sample was a result of an individual transfection paired with an eGFP transfection. Significant fold differences from corresponding control values (immuno-purified eGFP) are indicated by * (*p* < 0.05), ** (*p* < 0.005) and *** (*p* < 0.001).

To demonstrate that hCLCA1 was acting on its own and not in conjunction with other components secreted by the HEK293 cell line, immuno-purified hCLCA1 from FBS-free medium was tested. The presence and purity of hCLCA1 was confirmed with western blot and silver stain (Figure 6A). Since the concentration of immuno-purified hCLCA1 was beyond the detection range of Bradford protein assay (> 1 μg/mL), we measured the concentration of hCLCA1 using a standard curve generated by a 2-fold dilution series of lysozyme on a silver stained SDS-PAGE gel (Figure 6B). Densitometry analysis showed that the concentration of immuno-purified hCLCA1 was 6.2 ± 0.3 pg/μL (Figure 6C). Therefore, the maximum hCLCA1 concentration achievable for macrophage activation was 93.3 pg/mL. Macrophages dosed at 93.3 pg/mL of pure hCLCA1 activated similarly to that induced by the conditioned FBS-free medium after 48 h, although only IL-1β levels were enhanced after 24 h (Figure 6D and 6E).

**Figure 7:**
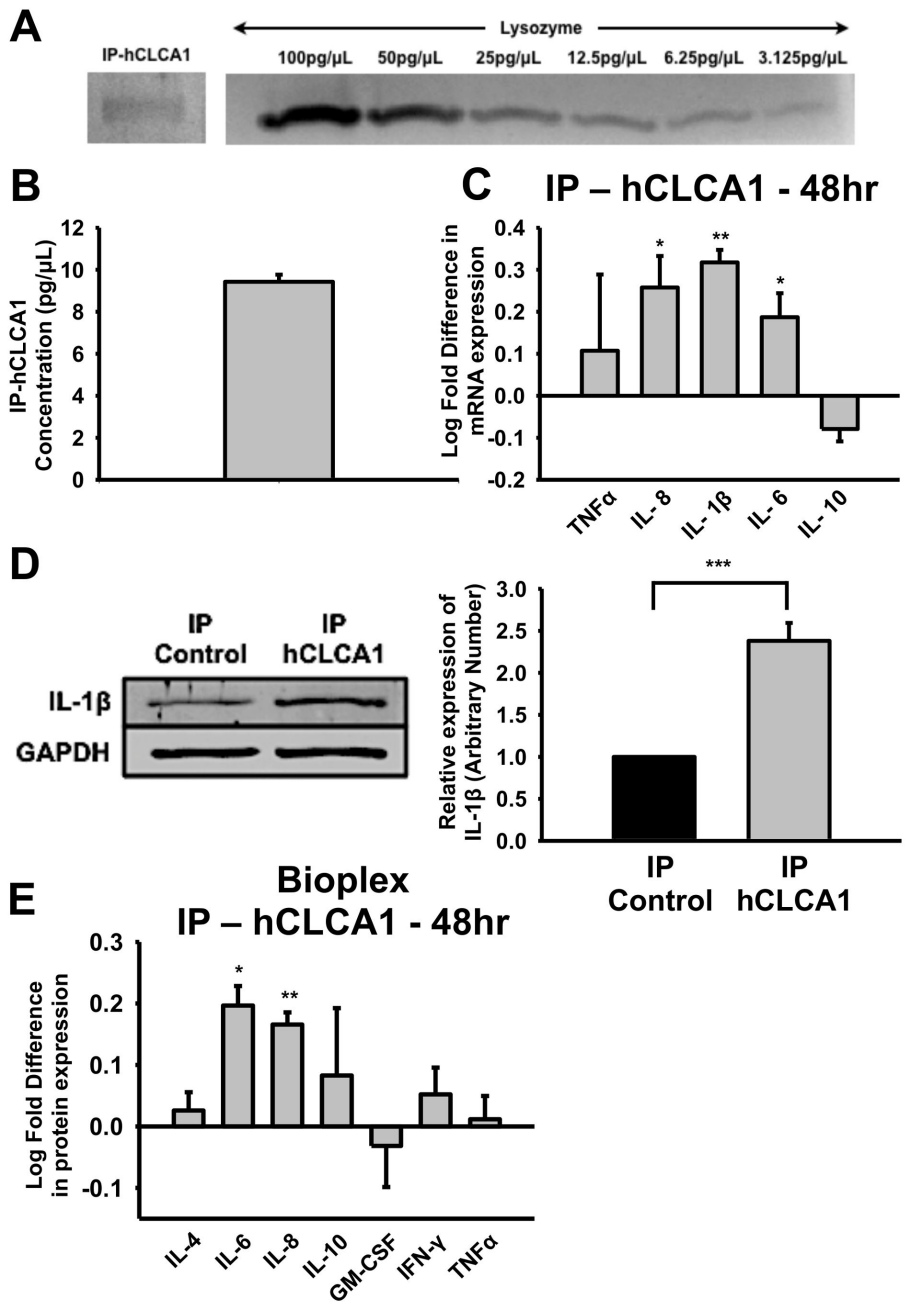
Enhanced macrophage activation with higher concentration of immuno-purified hCLCA1. (**A**) Representative silver stained SDS-PAGE gel showing immuno-purified hCLCA1 and a 2-fold dilution series of lysozyme. (**B**) Densitometry analysis using lysozyme standard curve demonstrated a higher concentration of pure hCLCA1 (9.425 ± 0.335 pg/μL) was immunoprecipitated with an optimized protocol. Result was presented as the mean of 3 samples ± SEM. (**C**) The mRNA expression of cytokines in macrophages stimulated with a higher concentration of hCLCA1 for 48 h was quantified using RT-qPCR. The fold difference was calculated against the corresponding control (immunoprecipitation of eGFP using hCLCA1-N14 antibody). Results were presented as the means of 4 samples ± SEM. (**D**) Representative Western blots showing intracellular IL-1β and GAPDH levels in immuno-purified eGFP or hCLCA1-stimulated macrophages. GAPDH was used as a loading control for densitometry analysis. Immuno-purified hCLCA1-stimulated macrophages had a 2.38 ± 0.21 folds increase in IL-1β protein levels over immuno-purified eGFP-stimulated macrophages (the hCLCA1-induced IL-1β was normalized to the eGFP-induced IL-1β in each sample). Results were presented as the means of 7 samples ± SEM. (**E**) Secreted cytokine protein expression in macrophages stimulated with a higher concentration of immuno-purified hCLCA1 was analyzed using Bio-plex Suspension Array System. The fold difference of each sample was compared against the corresponding control. Results were the means of 3 samples ± SEM. Significant fold differences from corresponding control values are indicated by * (*p* < 0.05), ** (*p* < 0.005) and *** (*p* < 0.001).

To demonstrate that higher concentration of pure hCLCA1 can elicit a stronger effect on macrophages, we optimized the immunoprecipitation protocol to yield a higher hCLCA1 concentration. The improvement resulted in the concentration of immuno-purified hCLCA1 as determined through densitometry analysis on a silver stained SDS-PAGE gel to be 9.4 ± 0.3 pg/μL (Figure 7A and 7B). This allowed macrophages to be activated with a maximum concentration of 141.7 pg/mL immuno-purified hCLCA1 for 48 h. Enhanced mRNA expression of pro-inflammatory cytokines was observed with increased hCLCA1 concentration (Figure 7C). For example, mRNA expression levels of IL-1β increased significantly from 0.178 log folds to 0.317 log folds (*p* < 0.05) with the increase in hCLCA1 concentration (Figure 6E and 7C). 

The increase in cytokine mRNA expression mentioned above was translated into an elevated cytokine protein levels. Western blot and densitometry analysis showed that intracellular IL-1β protein levels increased by 2.38 ± 0.21 folds (*p* < 0.001) over the control (Figure 7D). However, neither LPS nor hCLCA1-activated macrophages produced detectable extracellular IL-1β protein in the medium using western blot anaylsis (data not shown). Suggesting, IL-1β release is dependent on factors other than activation in this cell line. We then used a Bio-Plex suspension array system to measure the extracellular cytokine protein expression levels in the medium of control immuno-purified eGFP- and hCLCA1-activated macrophage. Significant fold increase over control protein levels of IL-6 and IL-8 was observed, while IL-4, IL-10, GM-CSF, IFN-γ, and TNF-α protein levels did not change significantly; and IL-2 was beyond the detection limit (Figure 7E). 

## Discussion

The results demonstrate that secreted hCLCA1 is able to function as a signaling molecule by activating both cell-line and primary porcine alveolar macrophages. Confirmation of the function of hCLCA1 as a signaling ligand for airway macrophages can begin to explain its pleiotropic effects [7].

### Type of response

The signaling function was initially demonstrated using conditioned FBS-containing medium from HEK293 cells heterologously expressing hCLCA1. At an optimal dose of 1 mg/mL, the conditioned FBS-containing medium activated macrophages over time similarly to the effect of LPS (Figure 2). To dose the macrophages without drastically changing the ionic and nutrient composition of the medium, the protein was concentrated out of the conditioned medium. However, this procedure also concentrated the FBS, which could have been deleterious to the macrophages at concentrations of 10 mg/mL, reducing the pro-inflammatory response (Figure 1A). Alternatively, the high concentrations of FBS and hCLCA1 together could turn off the pro-inflammatory response.

Interestingly, activating the macrophages with FBS-free hCLCA1 at higher concentrations of FBS (10%) in the growth media resulted in only a slight to moderate decrease in the pro-inflammatory response (Figure 3A). This decrease was not as significant as that of the 10 mg/mL (concentrated) hCLCA1 in FBS-containing medium (Figure 1A). This difference suggests that a high concentration of hCLCA1 itself may actually begin to dampen the pro-inflammatory response, a trend that was seen with FBS-free hCLCA1-conditioned medium when applied to a macrophage cell line (Figure 4). Evidence for higher concentrations of hCLCA1 having a reduced pro-inflammatory response was salient in the porcine alveolar macrophage experiment, where the strong pro-inflammatory response induced by low concentrations of FBS-free hCLCA1-containing medium was lost at higher concentrations (Figure 5).

Together, these results demonstrate that hCLCA1-containing medium has the capacity to induce a pro-inflammatory response that is dependent on its concentration, and the effect of activation is not cell-line dependent. In addition, these results also illustrated hCLCA1’s ability to activate macrophages within a mixed milieu of macromolecules. A strong pro-inflammatory response was observed when macrophages were activated with 1 mg/mL of FBS-containing hCLCA1 conditioned medium (Figure 2A). The FBS-containing hCLCA1 conditioned medium is comprised of a large number of immuno-reactive factors such as cytokines or growth factors. In pathophysiological settings, hCLCA1 would also have to function in such an environment of macromolecules and immuno-reactive factors presented in the BAL fluid of inflamed airways [26].

### Macrophage activation by pure hCLCA1

To confirm that macrophage activation is not dependent on constituents that might potentially be induced and secreted by hCLCA1 in HEK293 cells, we immunoprecipitated hCLCA1 from FBS-free hCLCA1-containing medium. In the initial experiment in which 93.3 pg/mL of hCLCA1 was used to activate macrophages, the response was limited to IL-1β in the first 24 h. Up to 48 h of macrophage activation was required for IL-8 to increase significantly as well (Figure 6D and 6E). It should be noted that 93.3 pg/mL of hCLCA1 is lower than the physiological concentration secreted from human airway epithelial cells [27], thus a weak response was expected. However, increasing the dose to 141.7 pg/mL of hCLCA1 elicited a stronger cytokine mRNA expression in macrophages at 48 h (Figure 7C), demonstrating pure hCLCA1’s ability to function in a dose dependent manner. This increase in cytokine mRNA expressions was sufficient to increase cytokine protein expression, further supporting the physiological relevance of these findings (Figure 7D and 7E). In fact, the concentration of hCLCA1 employed in this study is similar to physiological concentration of signaling molecules found in inflamed airway, as many cytokines are expressed in the pg/mL to ng/mL range in bronchoalveolar lavage (BAL) fluid in asthmatic patients [26,28,29]. 

### Potential activation mechanisms

Taken together, these findings indicate that airway macrophage activation is an intrinsic property of hCLCA1. hCLCA1 can elicit a dose-dependent response to macrophage activation. Generally, activation of pro-inflammatory response has a receptor-driven signal transduction mechanism and is dependent on ion-channel activation at the cell surface to proceed [16,18].

The protein hCLCA1 could also activate macrophages by ion-channel modulation. The ability of CLCA proteins to modulate multiple ion-channel types has been well documented [11,12,30]. These proteins increase single-channel conductance and directly interact with channel subunits [10,31]. However, inhibiting the hydrolase domain of CLCA proteins inhibited their capability to modulate calcium-activated chloride channels (CaCCs) [32]. Our findings suggest that since the hydrolase-inactive mutant could activate macrophages, then the CaCC-modulating ability of hCLCA1 is not involved in macrophage activation. However, such results would not preclude hCLCA1 being the modulator of other channels that are independent of its hydrolase domain. hClCA1’s Von Willebrand Factor-A (VWA) domain could for example act as a ligand to modulate ion channels. Such precedents exist as the α_2_δ subunit of the voltage gated calcium channel modulates its function by binding to an extracellular region of the channel pore subunit via its VWA domain and modifies its function [33]. 

Alternatively, hCLCA1 macrophage activation may occur through a signal transduction mechanism driven by a receptor ligand. Precedents exist, as some CLCA homologues contain novel integrin binding motifs [34,35]. However, the orthologs expressed in airway cells, including hCLCA1, do not possess this binding motif. Another domain that could potentially cause macrophage activation is the FN 3 domain in the C-terminus of the hCLCA1 protein. This FN 3 domain in fibronectin has been shown to induce cytokine expression in lung fibroblast [36]. However, increasing the concentration of the FN 3 domain in the medium by mutating the hydrolase domain did not increase macrophage activation (Figures 1A). 

### Pathophysiological implications

The signaling ability illustrated by our findings begins to explain how CLCA has such a pleiotropic effect on airway inflammation [7]. This effect was first demonstrated using airway adenoviral gene transfer expressing CLCA in BALB/c mice, in which the artificial expression drove and exacerbated mucus production, goblet cell metaplasia, eosinophil infiltration and airway hyper-responsiveness in an allergic asthmatic model [5]. However, subsequent studies in C57BL/6 and 129v mouse strain backgrounds produced both conflicting and corroborating results [7,37-39]. These conflicting studies could be explained if CLCA was functioning as a signaling protein which modulates a central mediator of the immune response (such as macrophages), which have a pleiotropic effect on lung inflammation. It is known that lung macrophages differ significantly between mouse strains, and such variations could explain the differences seen in the CLCA knockout models [5,7,37,39,40]. 

## Conclusions

**Figure 8:**
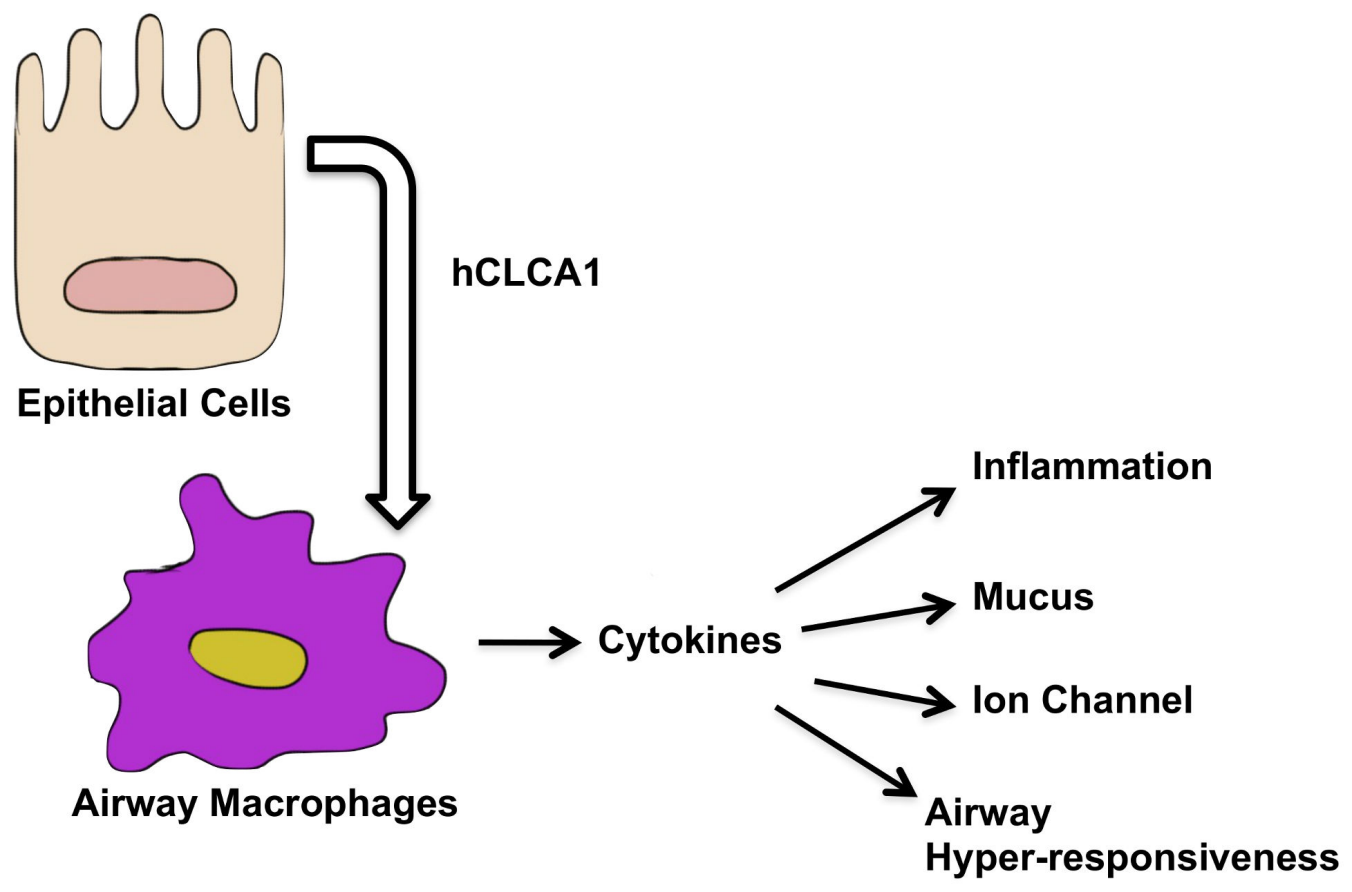
Schematic model of the effects of hCLCA1 on airway macrophages.

We have described the novel ability of secreted hCLCA1 to function as a signaling molecule that can activate airway macrophages. Such ability likely has a profound impact on the immune response in the airways, where the expression of this gene is massively up-regulated during inflammation [1]. Airway macrophages are pivotal regulators of the inflammatory response, and the ability of hCLCA1 to activate them could explain the pleotropic effect seen in airway inflammation models (where hCLCA1 is either over-expressed or knocked out). The cytokine responses of the macrophages would then modify the inflammatory response, mucus secretion, airway hyper-responsiveness and epithelial ion-channel function (Figure 8). Cytokines are known to modulate these processes [41,42]. Studies to identify the receptor and hCLCA1 domain containing the corresponding ligand are underway but are beyond the scope of this paper. The VWA domain could be one potential candidate, where the same domain in α2δ subunit binds to voltage-gated calcium channel [33]. The receptor and the ligand domain of hCLCA1 should be of therapeutic interest, as blocking either could have beneficial effects against airway inflammation. Furthermore, these findings open a new area of investigation into the function of CLCA proteins.
